# Distribution of CD4^pos^ -, CD8^pos^ – and Regulatory T Cells in the Upper and Lower Gastrointestinal Tract in Healthy Young Subjects

**DOI:** 10.1371/journal.pone.0080362

**Published:** 2013-11-12

**Authors:** Martin Tauschmann, Barbara Prietl, Gerlies Treiber, Gregor Gorkiewicz, Patrizia Kump, Christoph Högenauer, Thomas R. Pieber

**Affiliations:** 1 Division of Endocrinology and Metabolism, Department of Internal Medicine, Medical University of Graz, Graz, Austria; 2 Institute of Pathology, Medical University of Graz, Graz, Austria; 3 Division of Gastroenterology and Hepatology, Department of Internal Medicine, Medical University of Graz, Graz, Austria; Cincinnati Children's Hospital Medical Center, University of Cincinnati College of Medicine, United States of America

## Abstract

The gastrointestinal immune system is involved in the development of several autoimmune-mediated diseases, including inflammatory bowel disease, multiple sclerosis, and type 1 diabetes mellitus. Alterations in T-cell populations, especially regulatory T cells (Tregs), are often evident in patients suffering from these diseases. To be able to detect changes in T-cell populations in diseased tissue, it is crucial to investigate T-cell populations in healthy individuals, and to characterize their variation among different regions of the gastrointestinal (GI) tract. While limited data exist, quantitative data on biopsies systematically drawn from various regions of the GI tract are lacking, particularly in healthy young humans. In this report, we present the first systematic assessment of how T cells—including Tregs—are distributed in the gastrointestinal mucosa throughout the GI tract of healthy young humans by means of multi-parameter FACS analysis. Gastroduodenoscopy and colonoscopy were performed on 16 healthy volunteers aged between 18 and 32. Biopsies were drawn from seven GI regions, and were used to determine the frequencies of CD8^+^-, CD4^+^- and Tregs in the gastrointestinal mucosa by means of multi-parameter FACS analysis. Our data show that there is significant variation in the baseline T-cell landscape along the healthy human gastrointestinal tract, and that mucosal T-cell analyses from a single region should not be taken as representative of the entire gastrointestinal tract. We show that certain T-cell subsets in the gastrointestinal mucosa vary significantly among regions; most notably, that Tregs are enriched in the appendiceal orifice region and the ascending colon, and that CD8^pos^ T cells are enriched in the gastric mucosa.

## Introduction

The gut-associated lymphoid tissue (GALT) harbors the largest number of immune cells in the human body. It also represents the interface at which dietary antigens as well as microorganisms are recognized [1]. These signals from the environment are key to inducing immunological regulatory mechanisms and cooperation with the immune system to maintain intestinal homeostasis [2]. Imbalance in the equilibrium between intestinal microbes, intestinal epithelial cells and immune cells of the gut mucosa can lead to overwhelming immune stimulation, and to chronic inflammatory diseases of the gut, including inflammatory bowel disease (IBD) [3,4] and other autoimmune phenomena [1,5–7].

The suppression of such overwhelming immune stimulation is generally controlled by regulatory T cells (Tregs), a distinct CD4+ T cell population generated in the thymus and in the peripheral immune-organs (e.g. the GALT) [8,9]. Tregs have an inhibitory effect on proinflammatory cell populations and autoreactive effector cells. They exert their effector function by cell-cell contact-dependent mechanisms as well as mechanisms mediated by soluble factors (e.g., cytokine deprivation, CTLA-4 signaling, and interleukin (IL)-10 or transforming growth factor-β (TGF-β) production) [10]. Defects in the abundance and function of Tregs and resistance of effector T cells to Treg-mediated suppression contribute to failed T-cell regulation [11]. Such deficits of Tregs are often evident in patients suffering from autoimmune-mediated diseases, such as rheumatoid arthritis, systemic lupus erythematosus (SLE), multiple sclerosis, IBD, type 1 diabetes mellitus (T1DM) [11], and other inflammatory diseases of the intestine, such as necrotizing enterocolitis [12] and celiac disease (CD) [13,14].

Although intestinal and peripheral Tregs have been extensively studied in mice, those Tregs are characterized by the co-expression of the markers CD4 and Foxp3. In humans, however, phenotyping of Tregs is more complex. For this reason, a number of strategies have been described, including use of specific cell surface markers and biomarkers, to define and separate Tregs from other regulatory or effector T-cell subsets in humans [15–21]. The agreed definition of Tregs includes high expression of both CD25 and transcription factor forkhead box P3 (FoxP3) and low expression of IL-7 receptor (CD127) [11,17,19]. 

In humans, Tregs have largely been investigated in the peripheral blood, which may not accurately reflect the global number of Tregs in the body and in inflamed tissues. Data on Tregs at the site of inflammation, including the intestinal mucosa, are sparse, mostly due to difficulty in accessing the target organ. Nonetheless, there are a number of reports on the frequency of Tregs in the intestinal mucosa of people with inflammatory or autoimmune mediated diseases (e.g. IBD, T1DM) [17,22–24], and in healthy controls [25]. For example, Tregs in duodenal mucosa biopsy samples have been shown to be increased in active celiac disease (CD) [13,14] but are reduced in T1DM [22]. In IBD, Tregs are reported to be more frequent in inflamed mucosa, with frequencies being directly proportional to disease activity [24,26]. However, the data generated from those studies generally suffer at least one of three major drawbacks: 1. only a single region was examined, focusing on the degree of inflammation rather than on regional variations of T cell populations, whereas it is not known whether Tregs in any one region of the GI tract are representative of the whole GALT; 2. An immunohistochemical approach was used for Treg quantification in tissue, which relies on qualitative observations with low scoring reproducibility; and/or 3. Controls in studies investigating mucosal changes of the intestine are usually either symptomatic subjects with normal mucosa, or subjects over the age of 50 presenting for cancer screening. Until now, there have been no studies conducted in a well-defined, young, healthy population. Despite the fact that most autoimmune-mediated diseases have their peak incidence in younger patients, data on Tregs in the gastrointestinal mucosa have so far mostly been obtained from older subjects. Thus, quantitative data on biopsies systematically drawn from various regions of the GI tract are lacking, particularly in healthy young humans. 

In order to better understand how dysregulation of T-cell subtypes in the intestinal mucosa contributes to inflammatory and autoimmune conditions, it is crucial to investigate T-cell populations in healthy individuals, and to characterize their variation among different regions of the gastrointestinal tract. Such information may provide important clues as to which regions have significant roles in controlling immune regulation and GALT-associated autoimmune diseases. In this report, we present the first systematic assessment of how T cells—including Tregs—are distributed in the gastrointestinal mucosa throughout the GI tract of healthy young humans by means of multi-parameter FACS analysis. Our data shows that there is significant variation in the baseline T-cell landscape along the healthy human gastrointestinal tract. 

## Materials and Methods

### Ethics statement

The protocol was approved by the ethics committee at the Medical University of Graz, Austria, and performed in accordance with the Declaration of Helsinki and the principles of Good Clinical Practice. Patients gave written informed consent after the purpose, nature, and potential risks of the study were explained and before any study-related activities were started. 

### Research location and subjects

The study was conducted from January 2012 to August 2012 at the outpatient clinic, Department of Internal Medicine, Medical University of Graz (Austria). 16 apparently healthy subjects, all non-smokers, aged at least 18 years and with a BMI between 20 and 30 kg/m^2^ were included after an extensive screening procedure. The main exclusion criteria were any disease requiring medical treatment, presence of gastrointestinal symptoms, pregnancy, and participation in any interventional clinical trial. Subjects with abnormal laboratory findings of liver enzymes and kidney parameters, as determined at the screening visit, were excluded. All subjects reported that they had no allergic predispositions, no positive family history of autoimmune diseases or cardiovascular events in first degree relatives, and had had no recent infections or vaccinations.

### Study procedure

After the screening visit, eligible subjects underwent a gastroduodenoscopy and, on the following day, a colonoscopy. For bowel preparation, all subjects received bowel preparation with a polyethyleneglycol-based electrolyte solution (MOVIPREP®) as laxative. During the examinations, the subjects received sedation on request with midazolam and propofol according to local routine endoscopy procedures.

### Biopsy acquisition protocol

**Figure 1:**
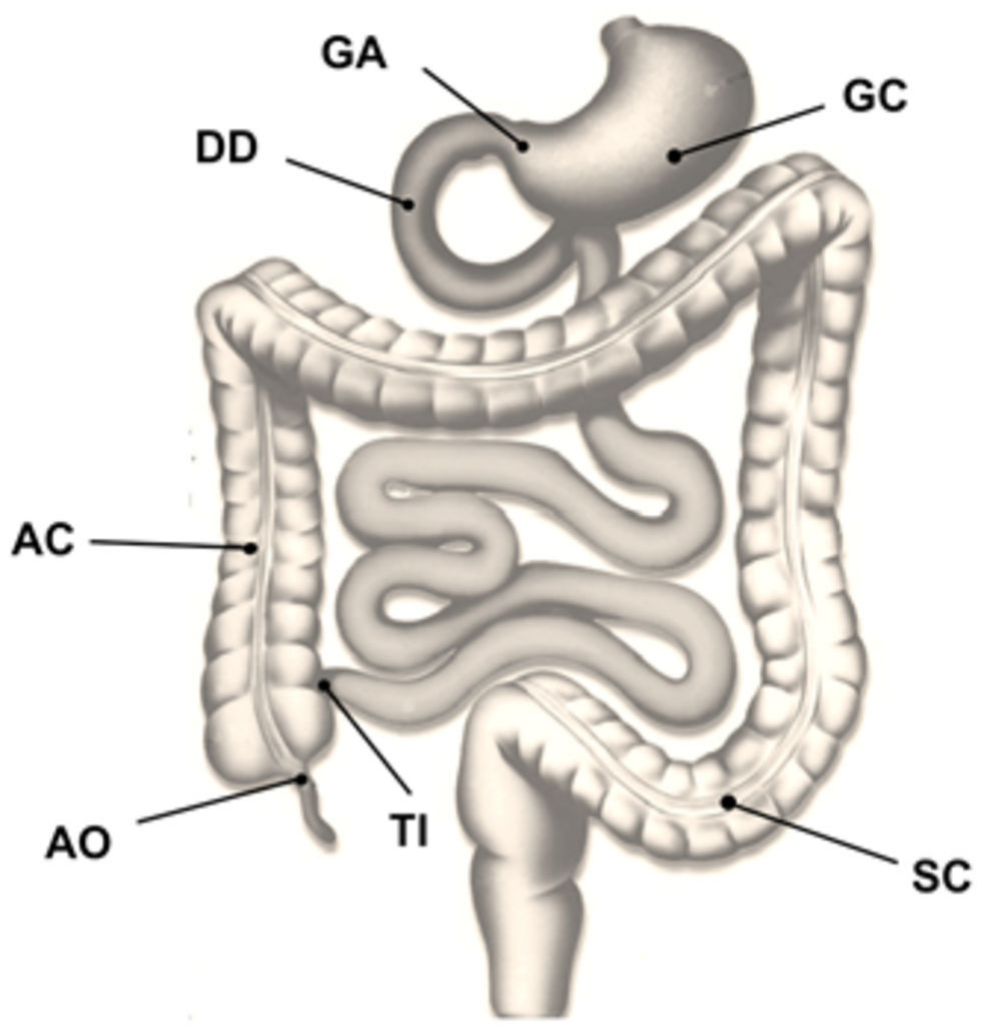
Schematic overview of biopsy regions including gastric corpus (GC), gastric antrum (GA), duodenum (DD), terminal ileum (TI), appendiceal orifice (AO), ascending colon (AC) and sigmoid colon (SC).

At gastroduodenoscopy, biopsies were taken from the gastric corpus (CC), the gastric antrum (GA) and the descending part of the duodenum (DD). At colonoscopy, biopsies were drawn from the terminal ileum (TI), the appendiceal orifice region (AO), the ascending colon (AC) 10 cm distal to the ileocecal valve and the sigmoid colon (SC) at 30 cm proximal to the anal canal (Figure 1). Three to six biopsies were taken from each region and immediately placed on ice in complete RPMI media containing 10% fetal calf serum (cRPMI-10), glutamine and penicillin/streptomycin (Life Technologies, Germany). Further analyses were started within one hour of the biopsy procedure.

### Histopathological assessment

Two biopsies were taken simultaneously from each GI location. The tissues were immediately fixed with formalin and embedded in paraffin to serve for H+E stained slides to be assessed by a pathologist. 

### Isolation of lamina propria mononuclear cells

The biopsies were transferred to a dithiothreitol/EDTA solution (Sigma, Germany and Life Technologies, Germany) and incubated for 15 minutes at 37°C. The tissue samples were then finely sliced and digested in a collagenase A solution (Roche, Germany) for 1 h at 37°C. After incubation, the cell suspension was passed through a 70-100 µm cell strainer (BD Biosciences, USA) and collected in a cRPMI-10 filled tube (Life Technologies, Germany). After centrifugation, the resulting pellet was resuspended in PBS (Life Technologies, Germany) and cell viability was confirmed by staining an aliquot with 0.4% trypan blue (Sigma, Germany) for microscopy.

### FACS staining

**Figure 2:**
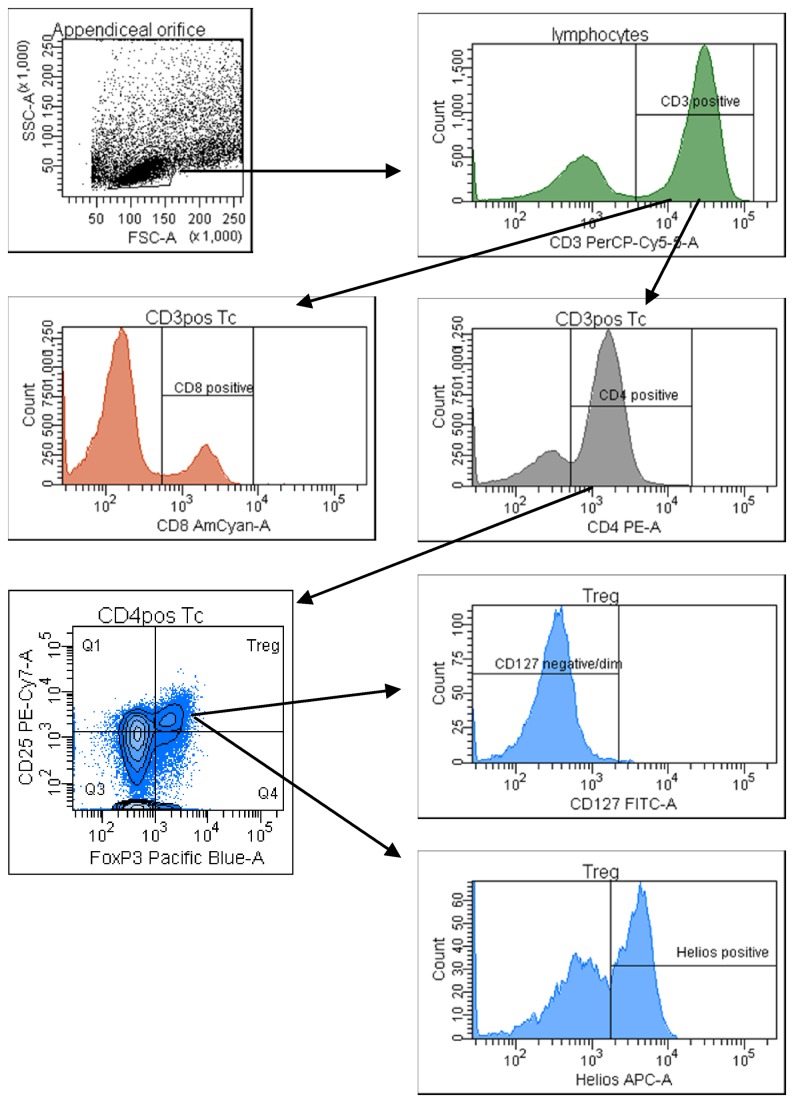
Representative gating strategy for the FACS analysis of lamina propria CD3+, CD4+, CD8+ and regulatory T-cells from pinch biopsies obtained from the appendiceal orifice region of one healthy subject.

The following fluorochrome-conjugated monoclonal antibodies were used for surface staining: anti-CD3 PerCp-Cy5.5, anti-CD4 PE, anti-CD8 V500, anti-CD25 PE-Cy7 and anti-CD127 FITC (BD Biosciences, USA). Cells were then fixed and permeabilized with a special buffer (BD Biosciences, USA). Intracellular staining for the transcription factor FOXP3 and the transcription factor Helios was performed by using anti-FoxP3 V450 (BD Biosciences, USA) and anti-Helios AF647 (eBiosciences, USA) monoclonal antibodies. Gates and quadrants were set based on isotype control staining and FMO-controls (Fluorescence Minus One) [27]. Data were acquired using a multicolor analysis on the FACSCanto II system (BD). As shown in a representative FACS plot in Figure 2, Tregs were characterized within the CD4 T-cell population by a high expression of both CD25 and FoxP3, and a low expression of IL-7 receptor (CD127) [17]. 

### Statistical analysis

Descriptive statistics are presented as mean ± standard deviation unless stated otherwise. Differences in the distribution of lymphocytes throughout the gastrointestinal system were analyzed using Friedman’s two-way analysis of variance by ranks. A p value of less than 0.05 was considered statistically significant and, when necessary, significance levels were adjusted using Bonferroni corrections for multiple comparisons. All statistical analyses were performed on the total study population (n=16) and a subgroup of subjects with *Helicobacter pylori* infection (n=4) using SPSS version 20 (PASW Statistics, USA). 

## Results

All 16 study participants completed the trial. Biopsies were taken from seven regions in each subject (Figure 1). The characteristics of our study population are described in Table 1. Endoscopic and histological findings are shown in Table 2, while T-cell subclasses and relative abundances in the intestinal mucosa are presented in Table 3.

**Table 1:** Demographic data and laboratory findings of our study population at baseline.

No. of patients	16
Females (%)	44
Age (yrs)	25 ± 4
Ethnicity	100% Caucasian
Height (cm)	172 ± 8
Weight (kg)	69 ± 11
Body mass index (kg/m^2^)	23 ± 3
Leucocytes (G/L)	6.2 ± 1.3 (normal range 4.4-11.3)
Serum C-reactive protein (mg/L)	3.8 ± 9.2 (normal up to -5.0)

Continuous variables are presented as means ± standard deviation (SD).

**Table 2:** Endoscopic findings and pathologic diagnosis at gastroduodenoscopy and colonoscopy.

	Variable	Frequency, n (%)
**Endoscopic findings**		
Gastroduodenoscopy	normal	9 (56.3)
	erythematous antral gastritis	4 (25.0)
	erythematous pangastritis	3 (18.8)
	erosive fundal gastritis	1 (6.3)
	erosive duodenitis	2 (12.5)
Colonoscopy	normal	13 (81.3)
	solitary polyp smaller than 1 cm	3 (18.8)
	Mass or tumor	0 (0)
**Histological findings**		
Gastroduodenoscopy	normal mucosa	9 (56.3)
	chronic gastritis	5 (31.3)
	*- H. pylori* associated	4 (25.0)
	chemical gastropathy	2 (12.5)
Colonoscopy	Biopsies of normal mucosa	16 (100.0)
	*- no signs of inflammation*	16 (100.0)
	Polyps	3 (18.8)
	*- Hyperplastic polyp*	1 (6.3)
	*- Juvenile polyp*	1 (6.3)
	*- Tubular adenoma*	1 (6.3)

**Table 3:** T-cell subclasses and relative abundances in the intestinal mucosa (n=16). Data are presented as Median (first quartile; third quartile).

	**GC**		**GA**		**DD**		**TI**		**AO**		**AC**		**SC**	
CD3pos (% of lymphocytes)	69.6	(54.0; 74.5)	53.3	(38.0; 63.0)	51.8	(44.0; 61.8)	59.1	(54.5; 70.6)	59.4	(53.0; 66.9)	60.7	(48.4; 64.4)	59.3	(53.0; 66.3)
CD8pos (% of CD3pos T cells)	39.7	(32.6; 52.9)	30.3	(22.6; 43.3)	17.8	(8.0; 25.4)	13.2	(9.9; 31.0)	13.0	(11.6; 16.7)	12.2	(9.2; 17.7)	12.1	(8.7; 17.5)
CD4 (% of CD3pos T cells)	17.7	(13.8; 27.4)	12.5	(9.1; 21.8)	36.1	(31.6; 41.5)	39.9	(35.1; 45.4)	38.2	(33.9; 46.7)	37.0	(34.6; 44.2)	41.2	(37.3; 46.2)
CD4/CD8 ratio	0.4	(0.3; 0.7)	0.4	(0.3; 0.8)	2.0	(1.3; 4.3)	3.0	(1.3; 4.1)	3.1	(2.5; 3.4)	3.2	(2.0; 3.9)	3.0	(2.4; 5.0)
Treg (% of CD4pos T cells)	2.0	(1.4; 3.3)	2.1	(1.2; 3.4)	1.6	(1.0; 3.2)	3.0	(2.5; 4.6)	4.6	(3.3; 5.7)	4.0	(3.2; 5.9)	3.5	(2.2; 5.2)

GC = gastric corpus, GA = gastric antrum, DD = duodenum, TI = terminal ileum, AO = appendiceal orifice, AC = ascending colon, SC = sigmoid colon

### CD4^pos^ and CD8^pos^ T cells are distributed differently among different GI locations

**Figure 3:**
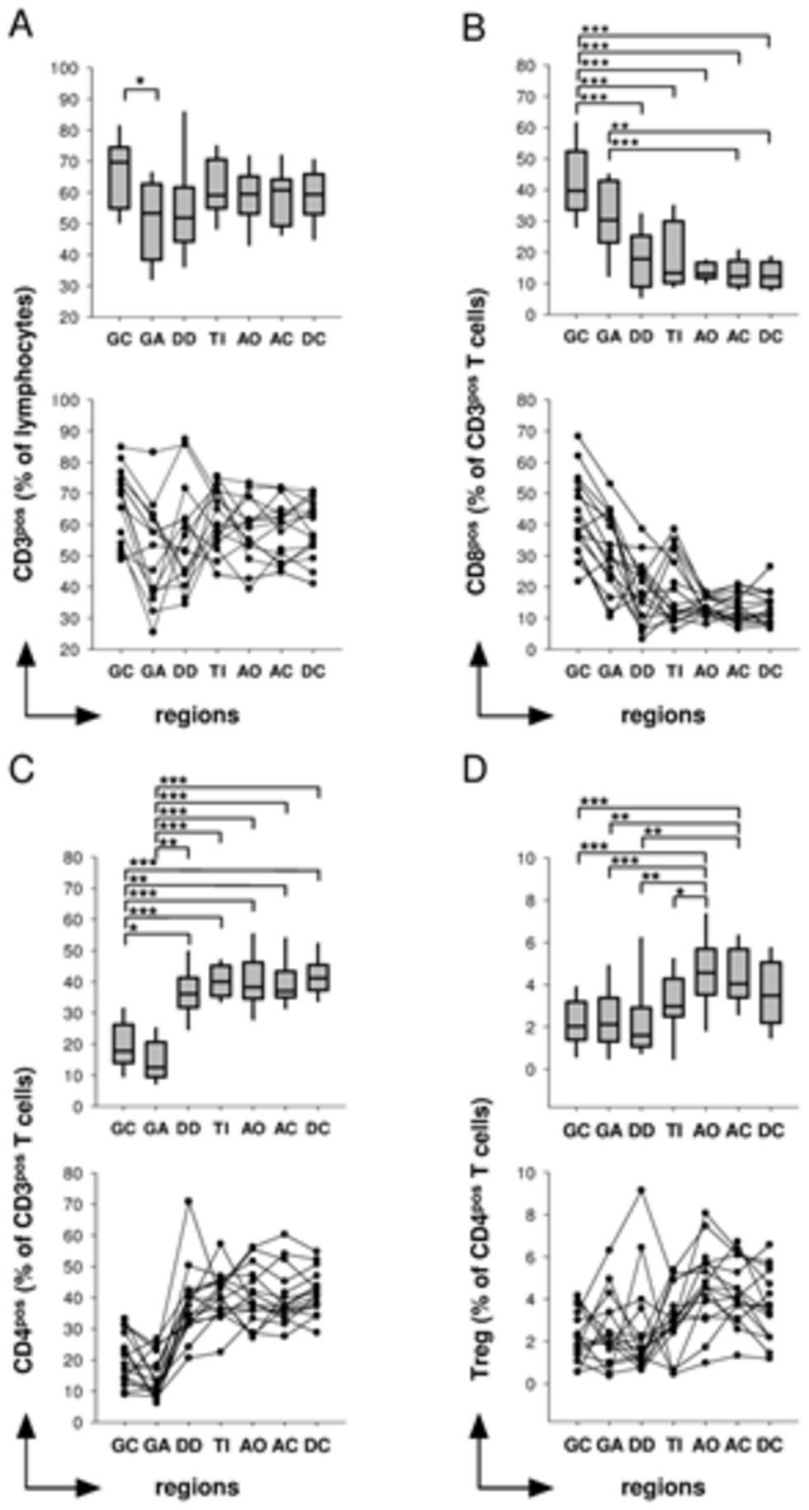
Relative abundance of CD3^pos^ cells, CD8^pos^ -, CD4^pos^ -T cells and Tregs in the intestinal mucosa. A) T cells are more or less evenly distributed in the gut B) CD8^pos^ T cells are enriched in the human gastric corpus and antrum. C) CD3^pos^CD4^pos^ T cells are predominantly found in the lower intestinal tract. D) The relative abundance of CD4^pos^CD25^high^CD127^low/neg^FOXP3 cells was highest in the appendiceal orifice region and the ascending colon. The figures represent cumulative flow cytometry data from all study participants (N=16) from all seven biopsy regions including the gastric corpus (GC), gastric antrum (GA), duodenum (DD), terminal ileum (TI), appendiceal orifice (AO), ascending colon (AC) and sigmoid colon (SC). Unless otherwise indicated, differences were not significant. *P<0.05; **P<0.01; ***P<0.001.

The frequency of T-cells among lymphocytes was quite consistent throughout the intestine (Figure 3A). A significant difference was only seen between the gastric corpus and gastric antrum (GC, median=69.6% vs. GA, 53.3%; adjusted p=0.006). In contrast, the distributions of T-cell subsets in the intestinal mucosa did vary significantly among gut regions (Figure 3 B,C,D, Table 3).

### CD8^pos^ T cells are enriched in the human gastric corpus and antrum

Levels of CD8^pos^ T cells among CD3^pos^ lymphocytes were significantly increased in the gastric mucosa (GC, median=39.7%; GA, 30.2%), particularly in the gastric corpus relative to the small (GC vs. DD, 17.8%, adjusted p= 0.001; GC vs. TI, 13.2%; adjusted p<0.001) and large intestine regions (GC vs. AO, 13.0%; adjusted p<0.001; GC vs. AC, 12.2%; adjusted p<0.001; GC vs. SC, 12.1%; adjusted p<0.001) (Figure 3B, Table 3). 

### CD4^pos^ T cells are predominantly found in the lower intestinal tract

The rate of CD4^pos^ T cells among CD3^pos^ lymphocytes increased along the gastrointestinal tract. Lowest levels were found in the gastric antrum (median= 12.5%) and the gastric corpus (17.7%). The relative abundance of CD4^pos^ cells increased significantly from the proximal to the distal parts of the intestinal tract, with maximal levels in the sigmoid colon (41.2%) (Figure 3C). Additionally, among the T-cell population, CD4^pos^ T cells outweighed CD8^pos^ T cells throughout all regions of the small and large intestine, as indicated by a CD4 to CD8 ratio above 1.00 (see Table 3).

### Tregs are augmented in the appendiceal orifice region and the ascending colon

The rate of CD4^pos^CD25^high^CD127^low/neg^FOXP3 cells among CD4^pos^ T cells was significantly higher in the appendiceal orifice region (median = 4.6%) and in the ascending colon (4.0%) than in the gastric region and duodenal mucosa (GC, 2.0%; GA, 2.1%; AO vs. GC; adjusted p<0.001; AO vs. GA, adjusted p=0.001; AC vs. GC; adjusted p=0.001; AC vs. GA; adjusted p=0.004; DD, 1.6%; AO vs. DD; adjusted p= 0.002; AC vs. DD; adjusted p=0.005) (Figure 3D). Furthermore, there is a significant difference between the frequencies of Tregs between the AO region and the terminal ileum (TI, 3.0%; AO vs. TI; adjusted p=0.039).

### Neither *Helicobacter pylori* nor gender alters T-cell distribution in the intestine

Four out of 16 subjects were found to be positive for *Helicobacter pylori* (HP), although all four showed no clinical symptoms or complications of HP gastritis. Histopathological assessment of simultaneous sampled biopsies revealed mild to moderate chronic gastritis in those subjects. There was no significant difference between the frequencies of T-cell subsets in the gastrointestinal mucosa in the HP-positive and the HP-negative groups (data not shown). Nor was there a sex-dependent difference in the distribution of mucosal T-cells in the intestine (data not shown).

## Discussion

We have systematically characterized the variation of intestinal mucosal T-cell populations across diverse compartments of the stomach and intestine in healthy young men and women for the first time. We show that certain T-cell subsets in the gastrointestinal mucosa vary significantly among regions; most notably, that Tregs are enriched in the appendiceal orifice region and the ascending colon, and that CD8^pos^ T cells are enriched in the gastric mucosa. Our results show that mucosal T-cell analyses from a single region cannot be taken as representative of the entire gastrointestinal tract.

The stomach is the first part of the gastrointestinal tract “seen” by food-derived luminal pathogens, and thus, it must provide an effective and lethal barrier to many of the microbes that enter the gut. Consequently, it presents a relatively hostile environment with fewer commensal bacteria [28] and therefore lower tolerance of the GALT to microbes. In this regard, the preponderance of CD8^pos^ T cells and low abundance of Tregs that we observe depict a rather cytotoxic milieu, which is in keeping with the major function of the stomach - to serve as a first line of defense.

Entering the small intestine, the relative abundance of CD4^pos^ cells in the duodenum is increased compared to the stomach, with duodenal CD4^pos^ cells now outweighing the CD8^pos^ T cells. This distribution pattern of high CD4^pos^ and low CD8^pos^ abundance is found throughout the small and large intestine, suggesting a key role for these regions in immune modulation. The number of commensal bacterial cells increases along the whole gastrointestinal tract [28], and there is an intimate crosstalk between these gut microbiota and the host immune system. Among other functions, the gut microbiota has an important role in the development of CD4^pos^ T cells [29]. This is supported by studies with germ-free mice, which show a marked decrease in the number of CD4^pos^ T cells in the lamina propria and an imbalance between the CD4^pos^ T cell subtypes like proinflammatory T helper 1- (Th1) and proregulatory Th2-cells [30,31]. Our results, demonstrating that CD4^pos^ T cells are enriched in the small and large intestine, are consistent with those observations.

The duodenal mucosa of patients with active celiac disease (CD) have higher frequencies of Tregs than the duodenal mucosa of treated CD and non-CD controls [13,14], as determined by FACS analysis. On the other hand, it has been reported that Tregs in the duodenum of T1DM patients were lower than in healthy subjects and patients with CD [22], suggesting that numerical deficits (T1DM) or functional deficits (CD and T1DM patients) of Tregs can arise in these autoimmune-mediated diseases. In those studies, the percentage of Tregs in the duodenum in control subjects was 6.8% [14] and 10% [22] of the total CD4^pos^ T cells. In our study, however, the frequencies of Tregs in the duodenum are considerably lower (1.6%). This discrepancy might be because our subjects were young healthy adults. It could also be because we used different gating strategies, subtly different FACS techniques or different antibodies for cell classification. Nonetheless, the choice of control group or the definition of healthy subjects in other studies is not always so carefully scrutinized. In the above-mentioned previous studies [13,14,22], all subjects underwent gastro-duodenal and colorectal endoscopy for diagnostic purposes. Controls were either children [22] or patients over the age of 50 [25] who either had preexisting gastrointestinal symptoms [13,22] or presented for cancer screening [25]. Allocation to the control group was post-intervention, based on normal endoscopic findings and histologically normal mucosa. Thus, it should be taken into account that co-morbidities and age might influence the frequency of Tregs [32], and that the attribute “healthy” may be misleading. 

The cecum and the appendix have been proposed to have a special immunological function, and could serve as a reservoir for commensal microbiota that facilitates re-inoculation of colonic bacteria after serious gut infections [33,34]. This would explain our findings of higher numbers of Tregs in the terminal ileum and appendiceal region, resulting in higher immune tolerance to bacterial antigens in these areas. This observation is also consistent with the observations of Wolff et al [25]. The assumption that the cecum and appendix could serve as a reservoir for commensal microbiota is supported by the high abundance of microbial biofilms in the appendix, and a high secretion of IgA and mucins in the cecum and appendiceal region [35,36]. Clinical observations support a role for the appendix in immunological and infectious intestinal diseases. Prior appendectomy in patients with ulcerative colitis has been shown to be associated with an improvement in clinical activity index or even complete remission [37–39]. On the other hand, in patients with clostridium difficile infection (CDI), prior appendectomy was positively associated with increased risk of disease recurrence [40]. Given the impact of the cecum and the appendix on microbiota balance, and the interplay between microbiota and the immune system, the greater abundance of Tregs in the mucosa of these regions is a significant finding, implying that future investigations into diseases involving intestinal Treg function should focus on these areas.

Data on T-cell frequencies in the mucosa of the large intestine largely derive from IBD studies [24,26,41,42]. In IBD, Tregs have been reported to be more frequent in the inflamed mucosa, with frequencies being directly proportional to disease activity [24,26]. However, in those studies, the exact biopsy location was not specified. Thus, a direct region to region comparison of frequencies is not possible. Regarding Treg counts in the colonic mucosa of the control group [24] or “healthy” subjects [25] in other studies, lower frequencies were found in the sigmoid compared with the ascending colon. Within the large intestine, our observations are in good agreement with those of Wolff et al. [25], confirming that T cell frequencies observed in the relatively easily accessible sigmoid colon do not represent T cell frequencies of other regions of the gut. 

The strengths of this study are that our subjects were healthy young men and women recruited and screened specifically for this study with no gastro-intestinal symptoms, and that the data are taken from seven regions of the gut. Thus, the data provide a representative picture of the healthy intestinal landscape, allowing region to region comparisons of T-cell populations in healthy subjects for the first time. The current study is a significant step towards understanding the variation of mucosal T-cell populations in distinct gut regions. Our findings support the assumption that each region of the gastrointestinal system has its own immunological specialization, and that the distributions of T cell subtypes between the stomach, the small intestine and the large intestine may reflect their different functions in immune regulation. These differences must be taken into consideration when performing mucosal biopsies from different parts of the intestinal tract in order to interpret study findings. 

A weakness of our study is that, due to the limited number of biopsies taken from each region, the investigation of other mucosal immune cells was precluded. The number of biopsies was limited in order to keep the procedure time low and avoid any potential risks that may arise from prolonged use of sedative drugs or from higher numbers of biopsies. In addition, our results are given as percentage of CD4^pos^ -, CD8^pos^ - and regulatory T-cells within CD3^pos^ T cells, since the absolute number of positive cells per microlitre cannot be calculated in intestinal biopsies using FACS technology. Another limitation is that, beyond the duodenum and the terminal ileum, other parts of the small intestine were not investigated in this study. This is because the small intestine is not accessible via routine endoscopic procedures that can be performed without danger for healthy volunteers. This uninvestigated region is assumed to have high immunological activity, according to its histopathological configuration, which includes large aggregated lymphoid follicles in the submucosa (e.g. Peyer’s Patches and the mesenteric lymph nodes) and the largest proportion of diffusely distributed populations of immune cells [43][44]. Whether T-cell populations in this area are significantly different from those in the duodenum and terminal ileum is yet to be determined.

It should be noted that, in our study, 4 out of 16 patients showed an asymptomatic *H. pylori* infection, which corresponds very well to the age-related prevalence of HP infection in the general population in Middle Europe and Austria [45–47]. Previous reports of T-cell distribution in the human gastric mucosa have revealed elevated numbers of T cells in the gastric mucosa of *H. pylori* infected patients, in particular in the Treg compartment [23,48–51]. However, we found no relationship between *H. pylori* infection and frequencies of T cells.

In view of the crucial role of mucosal T-cells in the balance between immune tolerance and immune defense, and in the development of autoimmune-mediated diseases, our data provide a reliable baseline for comparison with diseased intestines, in particular for future intervention studies.
